# Severe cases of seasonal influenza in Russia in 2017-2018

**DOI:** 10.1371/journal.pone.0220401

**Published:** 2019-07-29

**Authors:** Natalia P. Kolosova, Tatyana N. Ilyicheva, Alexey V. Danilenko, Julia A. Bulanovich, Svetlana V. Svyatchenko, Alexander G. Durymanov, Natalia I. Goncharova, Andrei S. Gudymo, Alexander N. Shvalov, Ivan M. Susloparov, Vasiliy Y. Marchenko, Tatyana V. Tregubchak, Elena V. Gavrilova, Rinat A. Maksyutov, Alexander B. Ryzhikov

**Affiliations:** 1 State Research Centre of Virology and Biotechnology “Vector”, Koltsovo, Novosibirsk, Russia; 2 Department of Natural Sciences, Novosibirsk State University, Novosibirsk, Russia; Icahn School of Medicine at Mount Sinai, UNITED STATES

## Abstract

The 2017–2018 influenza epidemic season in Russia was characterized by a relatively low morbidity and mortality. We evaluated herd immunity prior to the 2017–2018 influenza season in hemagglutination inhibition assay, and performed characterization of influenza viruses isolated from severe or fatal influenza cases and from influenza cases in people vaccinated in the fall of 2017. During the 2017–2018 epidemic season, 87 influenza A and B viruses were isolated and viruses of the 75 influenza cases, including selected viral isolates and viruses analyzed directly from the original clinical material, were genetically characterized. The analyzed A(H1N1)pdm09 viruses belonged to clade 6B.1, B/Yamagata-like viruses belonged to clade 3, and B/Victoria-like viruses belonged to clade 1A and they were antigenically similar to the corresponding vaccine strains. A(H3N2) viruses belonged to clade 3C.2a and were difficult to characterize antigenically and the analysis indicated antigenic differences from the corresponding egg-grown vaccine strain. The next generation sequencing revealed the presence of D222/G/N polymorphism in the hemagglutinin gene in 32% of the analyzed A(H1N1)pdm09 lethal cases. This study demonstrated the importance of monitoring D222G/N polymorphism, including detection of minor viral variants with the mutations, in the hemagglutinin gene of A(H1N1)pdm09 for epidemiological surveillance. One strain of influenza virus A(H1N1)pdm09 was resistant to oseltamivir and had the H275Y amino acid substitution in the NA protein. All other isolates were susceptible to NA inhibitors. Prior to the 2017–2018 epidemic season, 67.4 million people were vaccinated, which accounted for 46.6% of the country's population. Just before the epidemic season 33–47% and 24–30% of blood sera samples collected within the territory of Russia showed the presence of protective antibody titers against vaccine strains of influenza A and influenza B/Victoria-like, respectively. Mass vaccination of the population had evidently reduced the severity of the flu epidemic during the 2017–2018 influenza epidemic season in Russia.

## Introduction

Influenza is an infectious respiratory disease caused by influenza viruses type A, B, C and D. The disease symptoms range from mild, limited to the upper respiratory tract, to severe infection of the lower respiratory tract, which is fatal in some cases. Influenza virus infection can also lead to a wide range of non-respiratory complications affecting the heart, the central nervous system and other organs [1, 2].

Influenza virus commonly causes annual seasonal epidemics. An emergence in the human population of a variant of influenza A virus with new antigenic properties may cause a pandemic and the absence of pre-existing immunity in humans is often associated with increased severity of disease and increased mortality [3].

Thereby, monitoring population immunity and severe cases of influenza is an essential activity carried out within the framework of the Global Influenza Surveillance and Response System (GISRS), which will allow time to track the appearance of variants of the virus with modified virological, molecular-biological and/or antigenic properties.

We have been studying the population humoral immunity and the epidemic situation of influenza in Russia since 2009 [4–8]. This study continues our work on annual evaluation of herd immunity to influenza just before the influenza epidemic seasons in Russia and analyses of circulating influenza viruses in Russia, with a focus on the influenza viruses which cause severe illness, including cases with lethal outcome.

The epidemic rise in the incidence of influenza in Russia began during the 6th to 7th weeks of 2018, which is much later than usual and about two months later than in Western Europe and the US. In contrast to Western countries, the epidemic rise in the influenza disease incidence was smooth, and throughout the epidemic, the A(H1N1)pdm09, A(H3N2) and B viruses were detected in approximately equal proportions [9]. In general, the 2017–2018 epidemic season in Russia was characterized by a low influenza disease incidence in persons vaccinated in the fall of 2017 and the overall low mortality [10].

The purpose of this study was to assess population immunity to influenza viruses before the 2017–2018 Russian epidemic season and to characterize influenza viruses from clinical material from cases of severe influenza disease, autopsy material from influenza disease cases with lethal outcome, and clinical material from individuals vaccinated in the fall of 2017.

## Materials and methods

### Study of herd immunity

The study on blood sera testing was approved by the Ethics Committee IRB 00001360 affiliated with the Federal Budgetary Research Institution State Research Center of Virology and Biotechnology “Vector” (http://www.vector.nsc.ru/eticheskiy-komitet/). Blood samples were collected from donors who provided written informed consent on condition of anonymity. Sera were collected at the Sanitary-and-Epidemiological Centers of the Federal Service for Surveillance of Consumer Rights Protection and Human Wellbeing (Rospotrebnadzor, https://rospotrebnadzor.ru/region/structure/str_fguz.php) in 37 regions of Russia with 100–200 samples collected per region. The places of collection were located close to flyways and breeding grounds of wild waterfowl. Sera collection from healthy donors, transporting the clinical material to the State Research Center of Virology and Biotechnology “Vector”, Rospotrebnadzor (SRC VB “Vector”, Rospotrebnadzor) and the hemagglutination inhibition (HI) test were performed as previously described [6].

The A/Michigan/45/2015 A(H1N1)pdm09, A/HongKong/4801/2014 A(H3N2) and B/Brisbane/60/2008 (Victoria lineage) influenza viruses were kindly provided by the WHO Collaborating Center in Atlanta, US. The A/Anhui/01/2013 A(H7N9) virus was kindly provided by the WHO Collaborating Center in Beijing, China. The A/great crested grebe/Tyva/34/2016 A(H5N8) virus was isolated by the authors in Western Siberia in 2016 [11].

### Influenza virus isolation from nasopharyngeal swabs and autopsy material

The study of clinical material (nasopharyngeal swabs) and autopsy materials was approved by the Ethics Committee IRB 00001360 affiliated with the Federal Budgetary Research Institution State Research Center of Virology and Biotechnology “Vector” (http://www.vector.nsc.ru/eticheskiy-komitet/).

Samples were collected at the local Sanitary-and-Epidemiological Centers of the Federal Service for Surveillance of Consumer Rights Protection and Human Wellbeing (Rospotrebnadzor, https://rospotrebnadzor.ru/region/structure/str_fguz.php) after getting written, informed consent from the patients or their close relatives in accordance with the regulations of the Russian Federation. PCR-based diagnostics of the original material for influenza virus RNA was conducted in local laboratories, and then all the positive samples were sent to the SRC VB “Vector”, Rospotrebnadzor. All samples received in the SRC VB “Vector”, Rospotrebnadzor were retested using diagnostic PCR; and viral strains were isolated in MDCK by infecting a monolayer of cells [12]. Diagnostic real-time PCR for detecting RNA of influenza A virus and influenza B virus was performed using RNA isolated with the “RIBO-sorb” kit, cDNA prepared using the “RevertaL” kit, which was analyzed using the reagent kits “AmpliSense Influenza virus A/B-FL”, “AmpliSense Influenza virus H1N1pdm2009-FL” and “AmpliSense Influenza virus H3N2-FL” manufactured by the Central Research Institute of Epidemiology of the Federal Service for Surveillance of Consumer Rights Protection and Human Wellbeing (Moscow, Russia).

### Serological analysis of influenza viruses

Serological typing/subtyping of isolated influenza virus strains and studying their antigenic features were carried out in the HI test by using postinfectious ferret reference sera [12]. The ferret reference sera to the A/Michigan/45/2015 A(H1N1)pdm09, A/HongKong/4801/2014 A(H3N2), B/Brisbane/60/2008 (Victoria lineage), B/Phuket/3073/2013 (Yamagata lineage) influenza viruses were used for serological typing of influenza virus isolates. All reference sera were kindly furnished by the WHO Collaborating Center on Influenza in Atlanta, US.

### Sensitivity to anti-neuraminidase drugs

Susceptibility of influenza viruses to neuraminidase inhibitors oseltamivir and zanamivir was tested in fluorescent neuraminidase inhibition assay with 2’-(4-methylumbelliferyl)-α-D-N-acetylneuraminic acid (MUNANA) substrate according to the protocol recommended by the WHO [13].

### Sequence analysis of influenza viruses

Sequencing was carried out at the FBRI SRC VB “Vector”, Rospotrebnadzor. To determine nucleotide sequences of viral genes and genomes, viral RNA was isolated using the RIBO-sorb RNA/DNA Extraction Kit (InterLabService, Moscow, Russia) according to the manufacturer's instruction. Reverse transcription reactions were carried out with Uni12 primer [14] for type A viruses and Uni11 for type B viruses using the First Strand cDNA Synthesis Kit (Thermo Scientific, Lithuania) according to the manufacturer's instruction. PCR amplification of cDNA was done according to previously described protocols with modifications [14, 15, 16]. Detailed protocols are available upon request. Deep sequencing of amplicons covering complete genomes was performed on an Illumina MiSeq using the MiSeq reagent kit v3 (Illumina, San Diego, US). The full-length genomes were assembled by alignment of reads to known references with bwa-0.7.15 [17]. The obtained nucleotide sequences were deposited in the Global Initiative on Sharing All Influenza Data (GISAID) database. Phylogenetic analysis was performed using the maximum likelihood method based on the Hasegawa-Kishino-Yano model with 1000 bootstrap replications using MEGA 6.0 software (http://www.megasoftware.net/) [18]. For comparison, sequences of strains deposited in GISAID were used. Amino acid sequences of the isolated strains genomes were analyzed using FluSurver (http://flusurver.bii.a-star.edu.sg).

## Results

### Investigation of herd immunity

**Fig 1:**
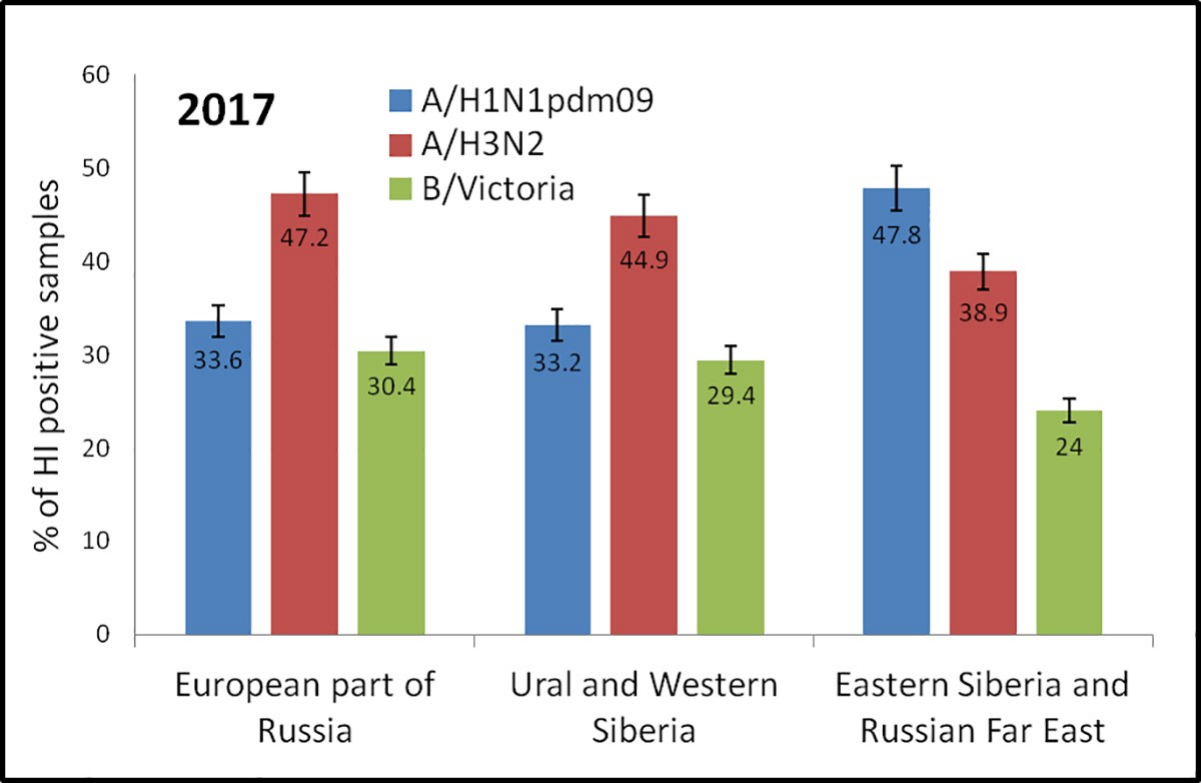
Seropositivity for influenza vaccine viruses in the studied groups of people from various geographical regions of Russia before the 2017–2018 epidemic season. The X axis–Russian regions, the Y axis–% of HI positive samples. The values on the bars are mean, the error bars denote the standard error of mean; Blue, positive samples to the A/Michigan/45/2015 A(H1N1)pdm09 vaccine virus; Red, positive samples to the A/HongKong/4801/2014 A(H3N2) vaccine virus; Green, positive samples to the B/Brisbane/60/2008 (Victoria lineage) vaccine virus.

To assess herd immunity to influenza A and B viruses, 3728 serum samples collected in the fall of 2017 from healthy donors living in the territory of the Russian Federation were examined. None of the samples reacted with antigens A(H5N8) and A(H7N9) in HI tests, even at a 1:10 dilution. Fig 1 depicts the results obtained in the HI tests with the seasonal influenza vaccine strains for 2017–2018.

More than a third of samples collected in different regions of Russia had significant titers (1:40 and above) in HI tests with vaccine strains of influenza A virus subtypes A(H1N1)pdm09 and A(H3N2) (Fig 1). The proportion of seropositive individuals for the influenza B virus of the Victoria genetic lineage was slightly lower. A comparison of the seromonitoring results with data obtained before the previous epidemic season [8] showed a slight decrease in population immunity in the population of the European part of the Russian Federation (50% of seropositive individuals in 2016, versus 34% in 2017) and in the Urals and Western Siberia (42% of seropositive individuals in 2016, versus 33% in 2017) to the influenza A(H1N1)pdm09 virus, while the proportion of seropositive individuals to A(H3N2) virus, on the contrary, increased in all regions (27–30% of seropositive individuals in 2016, versus 39–47% in 2017), which may be due to the prevalence of A(H3N2) subtype strains in circulation during the 2016–2017 season.

### Characterization of seasonal influenza viruses

In the epidemic season of 2017–2018, the SRC VB Vector Rospotrebnadzor received autopsy and clinical material from 61 regions of Russia. A total of 983 primary samples were received that were positive for RNA of influenza A and B viruses, including 90 samples of autopsy clinical material, 757 samples from patients with severe disease and 136 samples from patients vaccinated against influenza in the fall of 2017 (Table 1).

**Table 1:** Data on patients with cases of severe influenza disease, cases of influenza disease with fatal outcome, and patients vaccinated in the fall of 2017.

Group		Fatal cases (number)	Severe cases (number)	Vaccinated against influenza in the fall of 2017 (number of infected)
Sex				
	M	60	266	49
	F	30	438	72
	Unknown	-	53	15
Age group				
	0–18 years	14	232	81
	19–59 years	52	385	41
	60 years or older	22	70	11
	Unknown	2	70	3
Pregnant women		1	146	12
People vaccinated against influenza in the fall of 2017		4	132	136
Total		**90**	**757**	**136**

During the monitoring of seasonal influenza virus in the territory of the Russian Federation in the 2017–2018 epidemic season, 87 strains of influenza A and B viruses were isolated: 47 strains of A(H1N1)pdm09, 13 strains of A(H3N2), 25 strains of influenza B virus of the Yamagata lineage and 2 strains of influenza B virus of the Victoria lineage. Influenza viruses from 75 selected cases were sequenced and genetically characterized (including 31 cases, for which viral isolates were not available). Among them, 42 viruses were sequenced using the available viral isolates and 33 viruses were sequenced directly from clinical material (S1, S3 and S5 Tables).

#### Antigenic analysis of influenza viruses

The antigenic properties of all strains of influenza A(H1N1)pdm09 virus (including 17 isolates from lethal cases of the disease) were similar to the A/Michigan/45/2015 vaccine strain, strains of influenza B/Yamagata-like virus (3 of which were isolated from lethal cases of the disease) were similar to the B/Phuket/3073/2013 vaccine strain, and two strains of influenza B/Victoria-like virus were similar to the B/Brisbane/60/2008 vaccine strain.

Influenza A(H3N2) virus isolates had hemagglutination characteristics similar to the strains isolated in the previous season, namely: agglutination of guinea pig erythrocytes was detected only for the second passages of the strains in MDCK cell culture and only 7 of 13 isolates were able to hemagglutinate in the presence of 20 nM oseltamivir [8]. These particular strains were selected for the HI test. Reverse titers of anti-A/Hong Kong/4801/2014 serum (produced using the egg-based vaccine strain) in the HI with the A(H3N2) isolates were 8 times lower than the titer against homologous virus. Such a decrease in the reactivity of the antisera raised against the vaccine strain of egg-based origin against circulated A(H3N2) strains is noted by experts all over the world, while the analysis of viruses with the antisera raised against the cell-based vaccine strain did not reveal any significant antigenic differences from the vaccine strain, suggesting that egg adaptive changes decreased vaccine effectiveness [19].

#### Genetic analysis of influenza viruses

Influenza viruses from 75 selected cases (original specimen and/or MDCK isolate) were genetically analyzed and complete genomes or partial sequences of selected genes were obtained. Selected cases included 29 viruses of A(H1N1)pdm09 subtype (S1 Table), 26 viruses of A(H3N2) subtype (S3 Table), 18 influenza B/Yamagata-like viruses and 2 influenza B/Victoria-like viruses (S5 Table). The complete nucleotide sequences of the hemagglutinin (HA) gene for 70 influenza viruses were determined and were used to perform a phylogenetic analysis. The results of the phylogenetic analysis showed that the influenza A(H1N1)pdm09 viruses from 26 cases grouped in clade 6B.1 (S1 Fig), 25 influenza A(H3N2) viruses grouped in clade 3C.2a (of which 11 grouped in subclade 3C.2a1, 8 grouped in subclade 3C.2a2, and 6 grouped in subclade 3C.2a3) (S2 Fig), 17 B/Yamagata-like viruses—in clade 3 (S3 Fig) and 2 B/Victoria-like strains in clade 1A (S4 Fig).

A comparison of complete genomes (or partially sequenced genomes) of 29 selected influenza viruses A(H1N1)pdm09 showed a close genetic similarity with the A/Michigan/45/2015 vaccine strain (S1 and S2 Tables).

In total, A(H1N1)pdm09 cases comprised 65% of all studied and diagnostically confirmed influenza lethal cases in Russia, with H3N2 and influenza type B being 15% and 20%, respectively, according to data provided to SRC VB “Vector” by the local laboratories of Rospotrebnadzor Sanitary-and-Epidemiological Centers (Table 1). 29 cases of A(H1N1)pdm09 selected for this genetic study included 19 cases with lethal outcomes.

Analysis of the HA gene of 29 selected cases of A(H1N1)pdm09 influenza revealed in the major virus variant the presence of mutations D222G or D222N (H1 numbering) in the receptor-binding site in the MDCK isolates of four lethal cases out of 19 lethal cases included in the study (Table 2).

**Table 2:** D222G/N polymorphism detected in the HA gene of A(H1N1)pdm2009 viruses from lethal cases.

Strain	Passage history	D (HA 222)	G (HA 222)	N (HA 222)	Tissue	Pneumonia diagnosis
A/Samara/117868/2018	original	D Major (68%)	G Minor (26%)	-	lungs, trachea	no data
A/Samara/117868/2018	C1	D Minor (18%)	G Major (74%)	-	lungs, trachea	no data
A/Ulyanovsk/205/2018	C1	-	G Major (77%)	N Minor (17%)	lung	yes
A/Vladimir/314/2018	C1	-	G Major^np^	-	lung	yes
A/Irkutsk/1728/2018	original	D Minor (33%)	G Minor (27%)	N Major (37%)	trachea	yes
A/Irkutsk/1728/2018	C1	-	G Minor (19%)	N Major (79%)	lungs, trachea	yes
A/Krasnoyarsk/54/2018	original	D (70%)	G Minor (8%)	N Minor (18%)	bronchus	yes
A/Irkutsk/1727/2018	original	D Major (88%)	-	N Minor (8%)	lung	yes
A/Irkutsk/1727/2018	C1	D Major^np^	-	-	lungs, trachea	yes

Estimated proportion of mutations in HA based on sequence coverage of the corresponding codons is shown in parenthesis for the cases with detected polimorphysm, (-) amino acid not detected (values below detection limit)

^np^ no polymorphism detected.

Analysis of the HA D222G/N polymorphism using the next generation sequencing (NGS) data allowed us to detect and estimate the degree of the polymorphism in the studied cases. This analysis revealed the presence of D222G and D222N mutations in minor amounts in the primary clinical material of A/Krasnoyarsk/54/2018 lethal case (MDCK isolate was not available) and D222N mutation was detected in a minor amount in the primary clinical material of A/Irkutsk/1727/2018 lethal case (Table 2). The presence of D222N mutation was not detected in the viral isolate of A/Irkutsk/1727/2018 lethal case (Table 2). This difference from the primary material partially may be a result of using both lung and trachea original specimens to cultivate the viral isolate on MDCK cells compared to only lung specimen used for the direct sequencing of the original clinical material. This analysis also revealed the simultaneous presence of viruses with D222G and D222N mutations in three studied lethal cases (in two viral isolates, A/Ulyanovsk/205/2018 and A/Irkutsk/1728/2018, and in the original clinical material of A/Irkutsk/1728/2018 and A/Krasnoyarsk/54/2018 cases) (Table 2).

Analysis of the neuraminidase (NA) gene of 29 A(H1N1)pdm09 viruses revealed the presence of the H275Y mutation (H1 numbering) in a single virus, A/Samara/117868/2018 (in the primary material and in the MDCK isolate). The H275Y substitution was previously associated with highly reduced inhibition by oseltamivir and reduced or highly reduced inhibition by peramivir of A(H1N1)pdm09 viruses (according to the criteria of the WHO expert working group on surveillance of influenza antiviral susceptibility) [20, 21]. No mutations associated with resistance of influenza A(H1N1)pdm09 viruses to the NA inhibitor zanamivir were identified.

Genetic analysis of A(H3N2) influenza virus was performed for 26 selected cases, including 2 lethal cases (S3 Table). Based on the phylogenetic analysis of the 25 full-length HA sequences, the A(H3N2) viruses belonged to clade 3C.2a (S2 Fig), which could be further classified into three clades: 3C.2a1b, 3C.2a2 and 3C.2a3, according to the classification criteria presented in [22, 23]. For clade 3C.2a1b the combination of K92R and H311Q substitutions in HA1 was observed (H3 numbering is used for all A(H3N2) amino acid substitutions). Eleven viruses of the clade were further subgrouped. One of the subgroups included viruses with HA genes that encode E62G and R142G in HA1 with an additional substitution of T135K. Another subgroup included a virus with HA gene that encodes the T135N substitution. For 3C.2a2 clade, a combination of T131K, R142K and R261Q substitutions was seen, and a combination of N121K and S144K substitutions was observed for 3C.2a3 clade.

Analysis of amino acid substitutions in the HA of the studied influenza A(H3N2) viruses in comparison with the A/Hong Kong/4801/2014 vaccine strain revealed the presence of several mutations in antigenic site A in all studied viruses (S3 and S4 Tables). Most of these mutations were shown to be associated with antigenic drift. In addition, all studied viruses had mutations in antigenic site B, one of which, P194L, is in a site of antibody recognition and another mutation, K160T, was shown to be associated with antigenic drift. T160K reverse mutation was shown to be an adaptation mutation to the growth in embryonated chicken eggs [24] and it is absent in the cell-based A/HongKong/4801/2014 vaccine strain.

Amino acid substitutions in other studied A(H3N2) virus proteins were sporadic and not of substantive significance when compared to the A/Hong Kong/4801/2014 vaccine strain (S3 and S4 Tables).

Analysis of the NA of all studied A(H3N2) viruses for the presence of mutations conferring drug resistance revealed I222V amino acid substitution in the NA of A/Astrakhan/32/2017 strain. An association of this mutation with a slight increase in oseltamivir IC50 (less than 10 times) was shown for A(H1N1) [25]. No other mutations affecting NA inhibitors susceptibility were found.

A comparative genetic analysis of selected 18 B/Yamagata-like viruses, including 8 viruses from lethal cases (S5 Table), showed that all the viruses are genetically similar to the B/Phuket/3073/2013 vaccine strain. No mutations were identified that contribute to the increased pathogenicity of the viruses from lethal cases compared with the vaccine strain. The majority of the fatal of influenza B/Yamagata-like viruses were associated with the presence of the influenza illness risk group factors established by the WHO (age between 6 months to 5 years old, age more than 65 years old, chronic medical conditions) [26] (S5 Table).

Genetic analysis of 2 selected B/Victoria-like viruses showed that B/Kaliningrad/310/2018 and B/Kaliningrad/313/2018 strains are genetically similar to the B/Brisbane/60/2008 vaccine strain. No variants of influenza B/Victoria-like viruses with a deletion in the HA gene (in 162–163 or 162-163-164 amino acid positions in the protein) were found.

#### Analysis of influenza cases in vaccinated individuals

Of the 136 cases in individuals vaccinated with the trivalent vaccine, among those that recovered from influenza, 63 cases were diagnosed with influenza virus B, 41 with A(H3N2) virus, 25 with A(H1N1)pdm09 virus and 3 with influenza A virus that was not subtyped. There were only 4 fatal cases among vaccinated individuals and of these, 2 were diagnosed with A(H1N1)pdm09 virus and 2 with B/Yamagata-like virus.

One of the A(H1N1)pdm09 viruses from a lethal influenza case in a vaccinated individual, A/Irkutsk/1727/2018, was genetically analyzed. This analysis showed that this virus was similar in antigenic areas to the vaccine strain and the lethal outcome was associated with the presence of serious chronic diseases, which are considered as influenza risk factors by the WHO [26]. The second lethal A(H1N1)pdm09 influenza case in a vaccinated individual was associated with the presence of serious chronic diseases, one of which was chronic obstructive pulmonary disease (COPD).

#### Analysis of sensitivity to anti-neuraminidase drugs

In the course of the monitoring of seasonal influenza, sensitivity to oseltamivir and zanamivir of 87 isolated influenza viruses A and B was analyzed. According to the results of the phenotypic study, all isolated isolates of A(H3N2) and B influenza viruses were sensitive to anti-neuraminidase drugs oseltamivir and zanamivir. Among the 47 A(H1N1)pdm09 analyzed isolates, only one strain, A/Samara/117868/2018, was characterized by highly reduced sensitivity to oseltamivir with IC_50_ = 97.6 nМ and median value being 0.47 nМ for all studied strains falling within the normal sensitivity values range of 0.1–1.5 nM [13]. Susceptibility of this one oseltamivir-resistant strain to zanamivir was normal. Genetic analysis revealed the presence of the H275Y amino acid substitution in the NA of this strain, which is associated with resistance to oseltamivir of A(H1N1) pdm09 viruses.

## Discussion

In this study the assessment of population immunity to influenza viruses before the 2017–2018 Russian epidemic season was performed and a representative set of influenza viruses, which circulated in Russia during the 2017–2018 epidemic season, from cases of severe influenza disease, including cases with lethal outcome, and clinical material from individuals vaccinated in the fall of 2017 was serologically and genetically characterized.

In contrast to 2015–2016 and 2016–2017, when at the beginning of the epidemic period, one of the influenza A virus subtypes (A(H1N1)pdm09 and A(H3N2), respectively) prevailed in the northern hemisphere and influenza B viruses became prevalent at the end of the season, in 2017–2018, from the very beginning of the epidemic season, joint circulation of type A and B influenza strains was observed [6, 8].

The United States were characterized by a significant prevalence of influenza A viruses (71.2%) over influenza B viruses (28.8%); of the influenza A viruses, 84.9% belonged to the A(H3N2) subtype and 15.1% belonged to the A(H1N1)pdm09 subtype. Among influenza B viruses, viruses of the B/Yamagata genetic lineage prevailed at 88.8% [27]. For the majority of other countries in the Northern Hemisphere, a comparable distribution of the two types of viruses was observed with occurrences of a slightly higher prevalence of influenza A virus (Canada, 57%), or influenza B virus (Europe, 56%). Different countries in Europe demonstrated the predominance of a particular subtype of influenza A virus (A(H3N2) in the UK, A(H1N1)pdm09 in France, Germany, Italy); in the region as a whole, however, the proportions of both subtypes were approximately equal [9].

In China, influenza viruses of A and B type circulated in approximately equal proportion from the 46th week of 2017 to the 11th week of 2018. At the peak of the epidemic (2nd to 4th weeks of 2018), A(H1N1)pdm09 strains significantly prevailed among influenza A viruses [4TN].

Most of the influenza A(H1N1)pdm09 viruses studied in North America and Europe were antigenically similar to the A/Michigan/45/2015 vaccine strain and belonged to 6В.1 clade [27].

Most of the A(H3N2) strains characterized in the US belonged to 3C.2a clade and were antigenically similar to the A/Hong Kong/4801/2014 cell-based vaccine strain. However, only half of these strains were antigenically similar to the variant of the vaccine strain adapted to growth in embryonated chicken eggs and used for the vaccine production. These observed antigenic differences may be due to the occurrence of amino acid substitutions in the adaptation process. Similar antigenic characteristics were observed for A(H3N2) viruses in Europe where 3C.2a clade also was prevalent [19, 27].

In the 2018–2019 epidemic season, the WHO recommended replacing the H3N2 component of the vaccine with a new strain, A/Singapore/INFIMH-16-0019/2016. It was shown that antisera produced using the egg-based variant of this strain reacts better, though not entirely optimally, with strains that were prevalent in circulation in 2017–2018 [27].

In all countries of the Northern Hemisphere, the prevalent lineage among type B virus strains was B/Yamagata (89–98%). The B/Yamagata-like strains belonged to clade 3 and were antigenically similar to the B/Phuket/3073/2013 vaccine strain.

Among the B/Victoria-like viruses that were less common in the US and Europe, a significant proportion (80% in the US) had a deletion of six nucleotides in the HA gene. Such strains were first detected in the epidemic season of 2016–2017 [28]. These strains differ antigenically from the B/Brisbane/60/2008 vaccine strain, and therefore, a typical representative of the strains with the deletion, B/Colorado/06/2017-like virus, was included in the composition of the vaccine for the 2018–2019 season based on the recommendation of the WHO [29].

In Russia, none of the influenza virus subtypes was dominant in the 2017–2018 epidemic season: during the entire period, A(H1N1)pdm09, A(H3N2) and B/Yamagata-like viruses circulated in almost equal proportions. Few isolated cases of B/Victoria-like virus were detected and no variants of the virus with the deletion were identified [9, 30]. All virus strains of A(H1N1)pdm09, B/Yamagata-like, and B/Victoria-like isolated in this study were antigenically similar to the corresponding vaccine strains.

The A(H3N2) isolates analyzed in the HI test were antigenically different from the A/Hong Kong/4801/2014 egg-based vaccine strain, which may be explained by changes in its antigenic properties during the adaptation process for the growth in embryonated chicken eggs. According to the literature, serological studies showed that a significant proportion of A(H3N2) strains, which circulated in the world during the 2017–2018 epidemic season, were antigenically different from the A/Hong Kong/4801/2014 egg-based vaccine strain, while majority of the strains were antigenically similar to the cell-based vaccine strain [19, 27].

Analysis revealed an increase in the proportion of A(H1N1)pdm09 influenza virus detected in lethal influenza cases (65%) compared to equal proportions of A(H1N1)pdm09, A(H3N2) and influenza B viruses in all influenza cases. A similar increased proportion of A(H1N1)pdm09 compared with A(H3N2) detected in lethal cases compared to all cases was observed in Europe [31].

Molecular genetic analysis revealed the presence of D222G and D222N amino acid substitutions (H1 numbering) in the receptor-binding site of HA in the most predominant variant of the virus in four virus isolates from the 19 studied lethal cases of A(H1N1)pdm09 influenza (Table 2, S1 and S2 Tables). It is known that the presence of D222G and D222N mutations in the HA gene often correlates with increased disease severity and mortality. These mutations provide increased tropism of the virus to the cells of the lower parts of the human respiratory tract, which can lead to an increased risk of developing viral pneumonia. Some researchers suggest that increased virulence and lethality of A(H1N1)pdm09 influenza virus may be caused by the presence of D222G/N mutations in HA [6, 32].

It is suggested that these mutations accumulate as a result of selection from a mixture of influenza virus variants (quasi-species) in the lower parts of the respiratory tract where avian-like alpha-2,3 receptors with higher affinity for viruses with D222G/N mutations in the HA are more common compared to the upper respiratory tract. This selection occurs inside the infected organism, and at the stage of infection, D222G/N mutations are either absent or not dominant. This feature of D222G/N mutations highlights the critical importance of detecting the presence of minor quasi-species of the viruses with these mutations in clinical samples, since their presence can lead to selection of more pathogenic virus variants inside the body under favorable conditions [6, 32, 33]. In this study, analysis of the presence of polymorphism in the 222 position of the HA protein using NGS data revealed a minor presence of D222G/N mutations in the viruses from the primary material of two lethal cases of A(H1N1)pdm09 infection (Table 2).

Analysis of the presence of minor variants of the mutations revealed simultaneous presence of the virus variants with D222G and D222N mutations in three of the six cases examined (Table 2). The presence of multiple virus quasi-species in a viral population and their interaction with the immune system can lead to rapid adaptation and escape from immune control, as has been shown for primary HIV infection. These factors may possibly lead to a more severe disease [32].

For more precise data interpretation it is important to note that in our study, we observed 2–3 fold increase in quasispecies with D222G/N mutations in HA of two viral isolates obtained after one passage on MDCK cells compared to the original material, where these mutations were already present in significant quantities (Table 2). This increase in proportion of the mutations may have been caused by selection of the mutations due to receptor properties of MDCK cells [34]. Receptor specificity mutations such as D222G/N in the HA gene, which increase tropism to 2,3-linked sialic acid receptors, are known to be selected during virus cultivation in embryonated chicken eggs which have avian-like 2,3-linked sialic acid receptors in allantoic cells [35, 36, 37]. Cultivation of human influenza viruses on MDCK cells has proved to be a good alternative to egg inoculation. MDCK cells are easy to handle, reliable in maintaining genetic stability and are used as the standard cell line for influenza virus propagation [34]. MDCK cells have both 2,6- and 2,3-linked sialic acid receptors and due to this property may potentially favor selection of viral quasispecies that grow well in those cell types. In previously reported genetic stability studies of influenza virus propagation on MDCK cells, it was shown that receptor specificity mutations were not detected in viral isolates obtained after the first passage on MDCK cell [34, 36, 37], but that these mutations may arise through multiple passages [34]. Considering demonstrated genetic stability of the viral isolates after the first passage on MDCK cells, the presence of D222G/N mutations in major proportion in viral isolates obtained after one passage on MDCK cells, as was observed in our study, is indicative of the presence of the mutations in the original specimens, possibly in smaller proportions. Probability of the origin and speed of selection of D222G/N mutations in MDCK viral isolates needs to be further investigated in order to more precisely evaluate the significance of the observed genetic changes of seasonal influenza viruses in studies that employ virus isolation using MDCK cells.

For epidemiological analysis, it is important to determine the factors influencing transmissibility and selection of A(H1N1)pdm09 viruses with D222G/N mutations in humans. Selection of 222G/N virus variants can be influenced by virological properties as well as by the presence of risk factors in a person [6, 32].

Selection of D222G/N mutations inside an infected organism occurs due to the acquired increased ability of the virus to bind to α-2,3-type sialic acid receptors, which are present in the lower respiratory tract. However, in these mutant strains, the tropism to the cells of the human upper respiratory tract, where α-2,6-type sialic acid receptors are predominantly present, is weakened, and as a result, this may lead to reduced transmissibility of the mutant viruses among people. The reduced ability of the virus to spread through an infection of the upper respiratory tract may explain the low circulation of viruses with major variants of D222G/N mutations [6, 33].

Detecting D222G/N polymorphism, monitoring the increase in circulation of variants with D222G/N mutations and studying the evolution of A(H1N1)pdm09 viruses with D222G/N mutations, especially studying transmissibility, reassortment and the effect of genetic and virological properties of the virus on the pandemic potential, are necessary for timely identification of the emergence of potential pandemic threats in the circulating population of A(H1N1)pdm2009 viruses and are of particular importance for epidemiological analysis and prognosis.

Among the 87 analyzed isolates of influenza A and B viruses, only one A(H1N1)pdm09 strain isolated from a lethal case of influenza was resistant to oseltamivir while remaining susceptible to zanamivir; it contained the H275Y amino acid substitution in the NA protein. It is not known whether the patient was taking oseltamivir. Drug resistance can be a result of either selection in the viral population in patients taking anti-neuraminidase therapy or spontaneous mutation. In the epidemic season of 2017–2018, however, no more than 1% of the influenza viruses analyzed in the Northern Hemisphere were characterized by a decrease in sensitivity to NA inhibitors [27]. This class of influenza drugs is still highly effective and specific for the treatment of influenza and is recommended for use at an early stage of the disease.

In the Russian Federation, just before the 2017–2018 epidemic season, 67.4 million people were vaccinated, which accounted for 46.6% of the country's population. In the risk groups, the percentage of vaccinated individuals was even higher (89% of the employees of medical facilities, 85% of the employees of educational institutions, 70% of students (age 7 to 23 years), 63% of people over 60 years old) [10, 30]. It has been shown that in a fully mixed population, transmission could be interrupted if about 52% of the population was successfully immunized, assuming no mismatch between the vaccine strain and circulating strain [38]. The percentage of the population with protective antibody titers was 24%-47% against the influenza vaccine strains prior to the 2017–2018 epidemic season and it was similar to the assessment data obtained before the 2016–2017 epidemic season [8]. The lowest levels of protective population immunity were observed to B/Victoria-like vaccine virus (Fig 1). In the 2017–2018 epidemic season, the SRC VB “Vector”, Rospotrebnadzor received clinical samples from 136 cases of influenza among individuals vaccinated in the fall of 2017, 60% of which included cases of influenza disease among children under the age of 18 years old. These data may be partly explained by the suggestion that the protective antibody titers for children should be significantly higher than for adults (1:110 compared to 1:40) [39].

Undoubtedly, mass vaccination of the population had reduced the severity of the flu epidemic in Russia in the 2017–2018 season. The epidemic rise in the incidence of influenza began during the 6th to 7th weeks of 2018, the epidemic in the whole country lasted for 12 weeks, but in many regions, it ended in 5–6 weeks. In total, 10.4% of the population had influenza. The proportion of patients with severe acute respiratory infection of influenza etiology placed in the intensive care unit (2.3%) was less than in the previous season (5.4%) and was the minimum seen in the last 6 seasons [10].

In the trivalent vaccine recommended by the WHO for use in the influenza season of 2018–2019, 2 components were simultaneously replaced: A(H3N2) (A/Singapore/INFIMH-16-0019/2016 strain instead of A/Hong Kong/4801/2014 strain) and B/Victoria-like (B/Colorado/06/2017 strain of the new 1A.1 subclade instead of B/Brisbane/60/2008 strain) [27].

A new variant of B/Victoria-like influenza virus, which has a deletion in the HA gene, is antigenically different from the previously circulating strains. This variant has not spread in Russia yet, and therefore immunization before the 2018–2019 epidemic season was potentially very important for acquiring herd immunity to the new viral variant.

## Conclusions

Just before the 2017–2018 epidemic season in Russia 67.4 million people (46.6%) were vaccinated. The percentage of the population with protective antibody titers was 24%-47% against the vaccine strains prior to the 2017–2018 epidemic season. The rise of the influenza epidemic began during the 6th to 7th weeks of 2018 and the epidemic in the whole country lasted for 6 to 12 weeks depending on the region. In general, the 2017–2018 epidemic season in Russia was characterized by a low influenza disease incidence in persons vaccinated in the fall of 2017 and overall low mortality.

Throughout the epidemic, the A(H1N1)pdm09, A(H3N2) and B viruses were detected in approximately equal proportions. Among the analyzed isolated virus strains and viruses analyzed directly from the original clinical material and autopsy material, A(H1N1)pdm09 viruses belonged to clade 6B.1, A(H3N2) viruses belonged to clade 3C.2a, B/Yamagata-like strains belonged to clade 3, and B/Victoria-like strains belonged to clade 1A. All the viral isolates, except A(H3N2), were antigenically similar to the corresponding vaccine strains, according to the serological analysis. The analysis of A(H3N2) isolates indicated antigenic differences from the corresponding egg-grown vaccine strain.

The next generation sequencing of the original clinical material and isolated influenza strains of A(H1N1)pdm09 viruses revealed major or minor viral variants with HA containing D222G/N mutations, which were previously associated with the increased severity of the disease and mortality, in 32% of the analyzed lethal cases. This study demonstrated the importance of monitoring D222G/N polymorphism in the HA of A(H1N1)pdm09, including detection of minor virus variants with the mutations for epidemiological surveillance.

Of the 87 analyzed viral isolates only one strain of A(H1N1)pdm09 influenza virus was resistant to oseltamivir and had the H275Y amino acid substitution in the NA protein. All other isolates were susceptible to NA inhibitors.

Influenza vaccination and treatment with NA inhibitor drugs at the first manifestation of clinical signs of influenza disease are effective means of population protection against influenza.

## Supporting information

S1 Fig The phylogenetic tree for HA of A(H1N1)pdm09 influenza viruses analyzed in this study. (DOC)

S2 Fig The phylogenetic tree for HA of A(H3N2) influenza viruses analyzed in this study. (DOC)

S3 Fig The phylogenetic tree for HA of B/Yamagata-like influenza viruses analyzed in this study. (DOC)

S4 Fig The phylogenetic tree for HA of B/Victoria-like influenza viruses analyzed in this study. (DOC)

S1 Table Amino acid substitutions in genomes of A(H1N1)pdm2009 viruses in comparison with the A/Michigan/45/2015 vaccine strain. (DOC)

S2 Table Description of amino acid substitutions detected in A(H1N1pdm2009) viruses. (DOC)

S3 Table Amino acid substitutions in genomes of A(H3N2) viruses in comparison with the egg-based A/HongKong/4801/2014 vaccine strain. (DOC)

S4 Table Description of amino acid substitutions detected in A(H3N2) viruses. (DOC)

S5 Table Type B influenza virus cases characteristics. (DOC)

S6 Table Case characteristics and properties of influenza viruses isolated and characterized in the 2017–2018 epidemic season. (DOCX)
