# Correction: Severe cases of seasonal influenza in Russia in 2017-2018

**DOI:** 10.1371/journal.pone.0227382

**Published:** 2019-12-27

**Authors:** Natalia P. Kolosova, Tatyana N. Ilyicheva, Alexey V. Danilenko, Julia A. Bulanovich, Svetlana V. Svyatchenko, Alexander G. Durymanov, Natalia I. Goncharova, Andrei S. Gudymo, Alexander N. Shvalov, Ivan M. Susloparov, Vasiliy Y. Marchenko, Tatyana V. Tregubchak, Elena V. Gavrilova, Rinat A. Maksyutov, Alexander B. Ryzhikov

Rows 92–96 of the Virus column in S6 Table are incorrect. Please view the correct S6 Table below.

## Supporting information

S6 Table Case characteristics and properties of influenza viruses isolated and characterized in the 2017–2018 epidemic season. (DOCX)
