# Supplementary material for: Severe cases of seasonal influenza in Russia in 2017-2018
Source: PLoS One. 2019 Jul 29;14(7):e0220401. doi: 10.1371/journal.pone.0220401 (PMC6663013; doi:10.1371/journal.pone.0220401)
Supplement: S2 Fig — (DOC) [file pone.0220401.s002.doc]

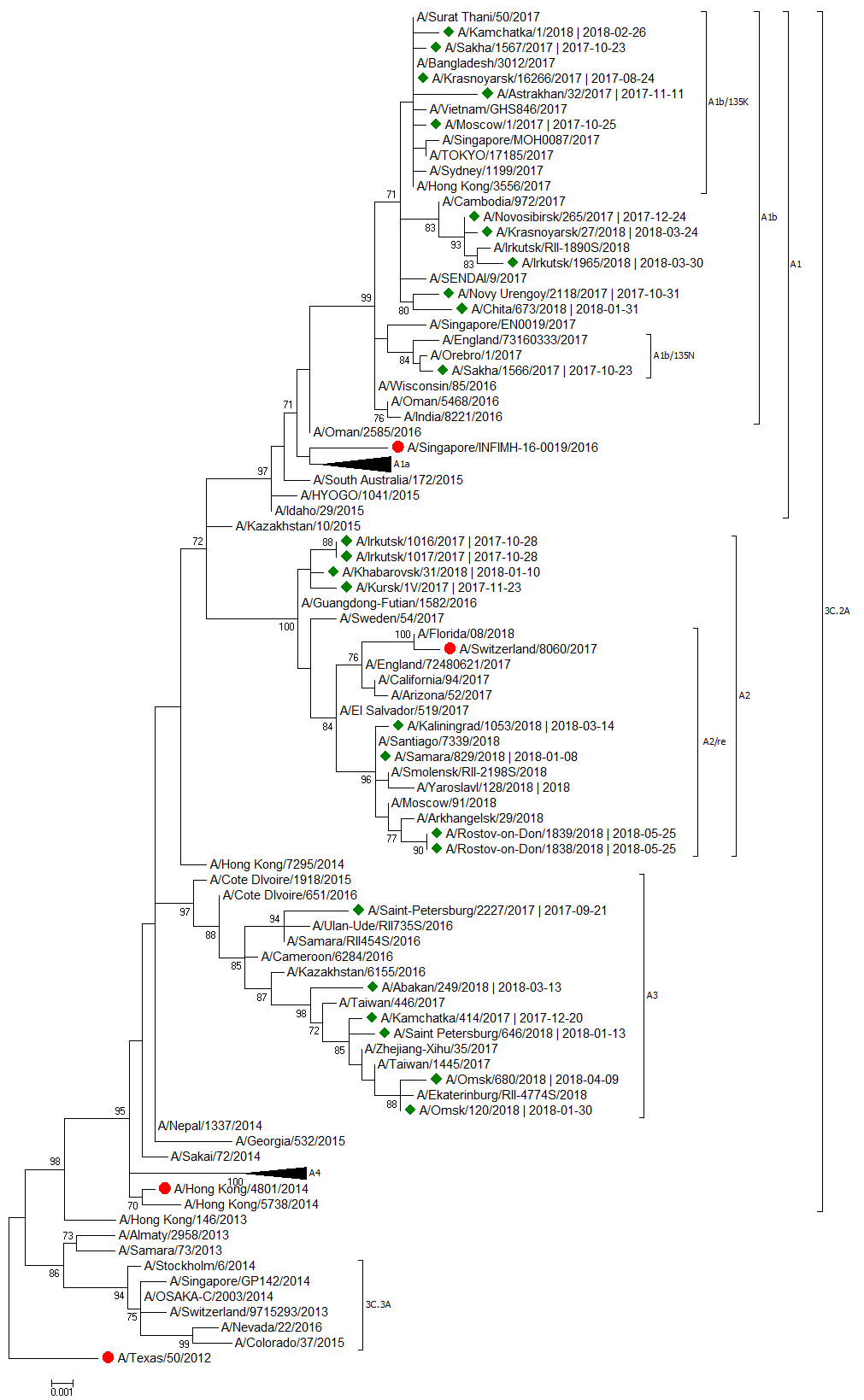


**S2 Fig. The phylogenetic tree for HA of А(H3N2) influenza viruses analyzed in this study.** Viruses isolated in Russia in the 2017-2018 epidemic season are indicated by green rhombi. Candidate Vaccine Viruses (CVV) are indicated by red circles. Scale bar indicates nucleotide substitutions per site.
