# Supplementary material for: Severe cases of seasonal influenza in Russia in 2017-2018
Source: PLoS One. 2019 Jul 29;14(7):e0220401. doi: 10.1371/journal.pone.0220401 (PMC6663013; doi:10.1371/journal.pone.0220401)
Supplement: S3 Fig — (DOC) [file pone.0220401.s003.doc]

**
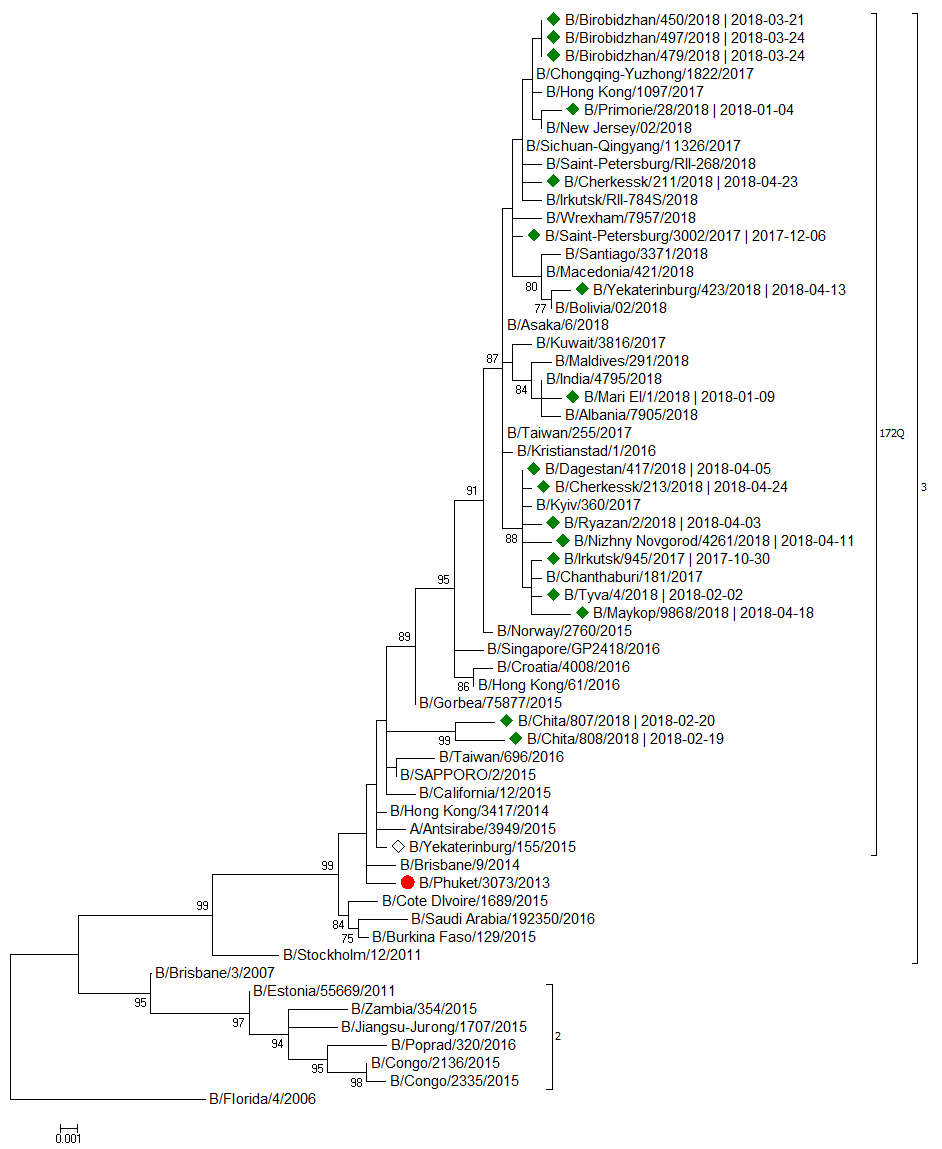
**

**S3 Fig. The phylogenetic tree for HA of B/Yamagata-like influenza viruses analyzed in this study.** Viruses isolated in Russia in the 2017-2018 epidemic season are indicated by green rhombi. A Candidate Vaccine Virus (CVV) is indicated by red circle. Scale bar indicates nucleotide substitutions per site.
