# Supplementary material for: Severe cases of seasonal influenza in Russia in 2017-2018
Source: PLoS One. 2019 Jul 29;14(7):e0220401. doi: 10.1371/journal.pone.0220401 (PMC6663013; doi:10.1371/journal.pone.0220401)
Supplement: S1 Table — (DOC) [file pone.0220401.s005.doc]

**S1 Table. Amino acid substitutions in genomes of A(H1N1)pdm2009 viruses in comparison with the A/Michigan/45/2015 vaccine strain.**

**The most prevalent sequence variant, deposited in GISAID, is shown for each virus; H1 numbering is used; amino acid substitutions detected in comparison with the vaccine strain are shaded; (-) in sequence analysis indicates that sequence data is not available; (-) in case characteristics data (pneumonia diagnosis, risk group, vaccination) indicates that data is not available; (*) indicates that MDCK isolate was not available. Analysis was done using FluSurver (**[**http://flusurver.bii.a-star.edu.sg**](http://flusurver.bii.a-star.edu.sg/)**).**

| Virus | Passage history | Collection date | Genome sequence | HA 68 | HA 137 | HA 163 | HA 183 | HA 222 | HA 223 | NA 222 | NA 275 | NA 365 | NS 87 | PA 57 | PA 268 | PA 400 | PB2 674 | Fatal case | Pneumonia diagnosis | Risk group (WHO) | Vaccination |
| --- | --- | --- | --- | --- | --- | --- | --- | --- | --- | --- | --- | --- | --- | --- | --- | --- | --- | --- | --- | --- | --- |
| A/Michigan/45/2015 | E3 | CVV |  | E | P | Q | S | D | R | N | H | I | S | R | L | P | A |  |  |  |  |
| A/Samara/117868/2018 | C1 | 12.01.2018 | complete | E | P | Q | S | G | Q | N | Y | I | S | R | L | P | A | yes | - | - | - |
| A/Ulyanovsk/205/2018 | C1 | 12.03.2018 | complete | E | P | Q | P | G | Q | N | H | I | S | R | L | P | A | yes | yes | yes | no |
| A/Vladimir/314/2018 | C1 | 17.04.2018 | complete | E | P | Q | P | G | Q | N | H | I | S | W | L | P | A | yes | yes | yes | no |
| A/Irkutsk/1728/2018 | C1 | 23.03.2018 | complete | E | P | Q | S | N | Q | N | H | I | S | R | L | P | A | yes | yes | - | no |
| A/Astrakhan/4/2018 | C1 | 27.02.2018 | complete | E | P | Q | S | D | Q | N | H | T | S | R | L | P | A | yes | yes | - | no |
| A/Kaliningrad/1146/2018 | C1 | 12.03.2018 | complete | E | P | Q | S | D | Q | N | H | I | S | R | L | P | A | yes | yes | - | - |
| A/Kaliningrad/3660/2018 | C1 | 12.03.2018 | complete | E | P | Q | S | D | Q | N | H | T | S | R | L | P | A | yes | yes | - | - |
| A/Irkutsk/1649/2018 | Original* | 16.03.2018 | partial | E | P | Q | S | D | Q | - | Н | - | S | - | - | L | A | yes | yes | - | no |
| A/Irkutsk/1648/2018 | Original* | 21.03.2018 | partial | - | P | Q | S | D | Q | - | Н | - | - | - | - | - | - | yes | yes | yes | no |
| A/Irkutsk/1727/2018 | C1 | 23.03.2018 | complete | E | P | Q | S | D | Q | N | H | I | S | R | L | P | A | yes | yes | yes | yes |
| A/Vladimir/263/2018 | C1 | 05.04.2018 | complete | E | P | Q | S | D | Q | N | H | I | S | Q | L | L | A | yes | yes | yes | no |
| A/Krasnoyarsk/54/2018 | Original* | 24.04.2018 | partial | E | S | R | P | D | Q | - | Н | - | S | R | L | P | A | yes | yes | - | no |
| A/Birobidzhan/1219/2018 | C1 | 10.05.2018 | complete | E | P | Q | S | D | Q | N | H | I | S | R | L | P | A | yes | yes | yes | - |
| A/Tomsk/6301/2018 | C1 | 02.04.2018 | complete | E | P | Q | S | D | Q | N | H | I | S | R | L | P | A | yes | - | - | - |
| A/Kemerovo/4/2018 | C1 | 06.03.2018 | complete | E | P | Q | S | D | Q | N | H | I | S | R | L | P | A | yes | yes | yes | no |
| A/Kaliningrad/3860/2018 | C1 | 14.03.2018 | complete | E | P | Q | S | D | Q | N | H | I | S | R | L | P | A | yes | yes | - | - |
| A/Yekaterinburg/472/2018 | C1 | 20.04.2018 | complete | D | P | Q | P | D | Q | N | H | I | S | R | L | P | A | yes | - | - | - |
| A/Yaroslavl/8/2018 | C1 | 28.03.2018 | complete | E | P | Q | P | D | Q | N | H | I | S | R | L | P | A | yes | - | - | - |
| A/Krasnoyarsk/8/2018 | C1 | 30.03.2018 | complete | E | P | Q | S | D | Q | N | H | I | S | R | I | P | A | yes | yes | - | no |
| A/Tyumen/1/2018 | C1 | 20.01.2018 | complete | E | P | Q | S | D | Q | N | H | I | P | R | L | P | A | no | - | - | yes |
| A/Astrakhan/9/2018 | C1 | 22.02.2018 | complete | E | P | Q | S | D | Q | N | H | T | S | R | L | P | A | no | - | yes | yes |
| A/Mykop/2353/2018 | C1 | 22.02.2018 | complete | E | P | Q | P | D | Q | D | H | I | S | R | L | P | A | no | - | yes | yes |
| A/Rostov-on-Don/3171/2017 | C1 | 20.09.2017 | complete | E | P | Q | S | D | Q | N | H | I | S | R | L | P | A | no | - | yes | - |
| A/Rostov-on-Don/3196/2017 | C1 | 22.09.2017 | complete | E | P | Q | S | D | Q | N | H | I | S | R | L | P | A | no | - | yes | - |
| A/Irkutsk/42/2017 | Original* | 11.12.2017 | partial | - | P | Q | S | D | Q | - | Н | I | - | - | - | - | - | no | - | yes | - |
| A/Samara/823/2018 | Original* | 02.01.2018 | partial | - | - | - | P | D | Q | N | H | I | S | R | L | P | - | no | - | - | - |
| A/Irkutsk/1615/2018 | C1 | 19.03.2018 | complete | E | P | Q | S | D | Q | N | H | T | S | R | L | P | A | no | yes | yes | no |
| A/Dagestan/602/2018 | C1 | 18.04.2018 | complete | E | P | Q | S | D | Q | N | H | T | S | R | L | P | A | no | yes | - | no |
| A/Krasnodar/421/2017 | C1 | 15.12.2017 | complete | E | P | Q | S | D | Q | N | H | I | S | R | L | L | E | no | - | - | no |
