# Supplementary material for: Severe cases of seasonal influenza in Russia in 2017-2018
Source: PLoS One. 2019 Jul 29;14(7):e0220401. doi: 10.1371/journal.pone.0220401 (PMC6663013; doi:10.1371/journal.pone.0220401)
Supplement: S2 Table — (DOC) [file pone.0220401.s006.doc]

**S2 Table. Description of amino acid substitutions detected in A(H1N1pdm2009) viruses.**

H1 numbering is used. Analysis was done using FluSurver ([http://flusurver.bii.a-star.edu.sg](http://flusurver.bii.a-star.edu.sg/)).

| Gen | Mutation | Description |
| --- | --- | --- |
| HA | E68D, P137S, Q163R, S183P | Antigenic drift. |
| HA | D222G/N | Mutations D222G/N were associated with a higher specificity of HA to avian-type receptors and tropism to the cells of the lower parts of the human respiratory tract. It was often correlated with severe desease and lethality. |
| HA | R223Q | Antigenic drift. A reverese mutation Q223R was associated with changes in HA receptor specificity toward avian-type receptors and is considered adaptive for virus growth in embryonated chicken eggs. |
| NA | N222D | Antigenic drift. |
| NA | H275Y | Strong drug resistance to oseltamivir and medium drug resistance to peramivir. |
| NA | I365T | Antigenic drift, virulence. |
| NS | S87P | Host specificity shift (statistical, S – human, P – avian). |
| PA | R57Q, R57W | Host specificity shift (statistical, Q – human, R – avian). |
| PA | L268I | Host specificity shift (statistical, I – human, L – avian). |
| PA | P400L | Host specificity shift (statistical, L – human, S – avian). |
| PB2 | A674E | Host specificity shift (statistical, T – human, A – avian). |
