# Supplementary material for: Severe cases of seasonal influenza in Russia in 2017-2018
Source: PLoS One. 2019 Jul 29;14(7):e0220401. doi: 10.1371/journal.pone.0220401 (PMC6663013; doi:10.1371/journal.pone.0220401)
Supplement: S3 Table — (DOC) [file pone.0220401.s007.doc]

**S3 Table. Amino acid substitutions in genomes of A(H3N2) viruses in comparison with the egg-based A/HongKong/4801/2014 vaccine strain.**

**The most prevalent sequence variant, deposited in GISAID, is shown for each virus; H3 numbering is used; amino acid substitutions detected in comparison with the vaccine strain are shaded; (-) in sequence analysis indicates that sequence data is not available; (-) in case characteristics data (pneumonia diagnosis, risk group, vaccination) indicates that data is not available; (*) indicates that MDCK isolate was not available. Analysis was done using FluSurver (**[**http://flusurver.bii.a-star.edu.sg**](http://flusurver.bii.a-star.edu.sg/)**).**

| Virus | Passage history | Collection date | Genome sequence | HA 122 | HA 128 | HA 131 | HA 135 | HA 142 | HA 144 | HA 160 | HA 193 | HA 194 | NA 150 | NA 221 | NA 222 | NA 245 | NA 247 | NA 329 | PB 2 63 | PB2 588 | PB1 375 | PA 57 | PA 266 | NS1 87 | NS1 221 | NS1 225 | NS1 227 | Fatal | Pneumonia | Risk group (WHO) | Vaccination |
| --- | --- | --- | --- | --- | --- | --- | --- | --- | --- | --- | --- | --- | --- | --- | --- | --- | --- | --- | --- | --- | --- | --- | --- | --- | --- | --- | --- | --- | --- | --- | --- |
| A/HongKong/4801/2014 | E5/E2 | CVV |  | N | T | T | T | R | S | K | F | P | R | D | I | S | S | N | V | I | S | Q | R | S | E | T | R |  |  |  |  |
| A/Chita/673/2018 | Original* | 31.01.2018 | partial | N | T | T | T | G | S | T | F | L | R | D | I | N | T | S | - | T | S | - | R | S | K | T | R | yes | yes | - | no |
| A/Omsk/680/2018 | C1 | 09.04.2018 | complete | N | T | T | K | G | K | T | F | L | R | D | I | N | T | N | I | T | S | Q | R | S | E | A | R | yes | yes | yes | no |
| A/Sakha/1566/2017 | Original* | 23.10.2017 | partial | N | T | T | N | R | S | T | F | L | S | D | I | N | T | S | - | - | S | Q | R | S | E | T | R | no | - | yes | yes |
| A/Saint Petersburg/646/2018 | C1 | 13.01.2018 | complete | N | T | T | K | G | K | T | F | L | R | D | I | N | T | N | I | T | S | Q | G | S | E | T | R | no | - | - | yes |
| A/Kaliningrad/1053/2018 | C1 | 14.03.2018 | complete | N | T | K | T | K | R | T | F | L | R | D | I | N | T | S | I | T | N | Q | R | P | E | T | R | no | - | - | yes |
| A/Abakan/249/2018 | C1 | 13.03.2018 | complete | N | T | T | K | R | K | T | F | L | R | D | I | N | T | N | I | T | S | Q | R | S | E | T | R | no | - | - | yes |
| A/Krasnoyarsk/16266/2017 | Original | 24.08.2017 | partial | N | T | T | K | G | S | T | F | L | R | D | I | N | T | S | I | T | S | Q | R | S | E | T | R | no | - | - | - |
| A/Nizhny Novgorod/8320/2017 | Original* | 21.09.2017 | partial | N | T | T | N | R | S | T | F | L | R | D | I | N | T | S | - | - | - | - | - | S | E | T | R | no | - | - | no |
| A/Saint-Petersburg/2227/201 | Original* | 21.09.2017 | partial | D | T | T | T | R | K | T | F | L | R | D | I | N | T | N | - | T | S | Q | R | S | E | T | R | no | - | - | no |
| A/Sakha/1567/2017 | Original* | 23.10.2017 | partial | N | T | T | K | G | S | T | F | L | - | - | - | N | T | S | - | - | - | - | - | - | - | - | - | no | - | yes | no |
| A/Moscow/1/2017 | Original* | 25.10.2017 | complete | N | A | T | K | G | S | T | F | L | R | N | I | N | T | S | I | T | S | Q | R | S | E | T | R | no | - | - | no |
| A/Irkutsk/1016/2017 | Original* | 28.10.2017 | partial | N | T | K | T | K | S | T | S | L | R | D | I | N | T | N | - | - | S | Q | R | S | E | T | K | no | yes | yes | - |
| A/Irkutsk/1017/2017 | Original* | 28.10.2017 | partial | N | T | K | T | K | S | T | S | L | R | D | I | N | T | N | - | - | - | - | - | - | - | - | - | no | - | yes | - |
| A/Novy Urengoy/2118/2017 | Original* | 31.10.2017 | partial | N | T | T | T | G | S | T | F | L | - | D | I | N | T | S | - | - | - | - | R | - | - | - | - | no | - | - | no |
| A/Astrakhan/32/2017 | C1 | 08.11.2017 | complete | N | T | T | K | G | S | T | F | L | R | D | V | N | T | S | I | T | S | Q | R | S | E | T | R | no | - | yes | no |
| A/Kursk/1V/2017 | C1 | 23.11.2017 | complete | N | T | K | T | K | S | T | F | L | R | D | I | N | T | N | I | T | S | Q | R | S | E | T | R | no | - | - | no |
| A/Kamchatka/414/2017 | Original* | 20.12.2017 | complete | N | T | T | K | G | K | T | F | L | R | D | I | N | T | N | I | T | S | Q | R | S | E | T | R | no | - | yes | no |
| A/Novosibirsk/265/2017 | Original | 24.12.2017 | partial | N | T | T | K | G | S | T | F | L | R | D | I | N | T | S | I | T | S | Q | R | S | E | T | R | no | - | - | no |
| A/Samara/829/2018 | Original* | 08.01.2018 | partial | N | T | K | T | K | R | T | F | L | - | - | - | N | T | S | - | - | - | - | - | - | - | - | - | no | - | - | - |
| A/Khabarovsk/31/2018 | Original* | 10.01.2018 | partial | N | T | K | T | K | S | T | F | L | - | - | - | - | - | - | - | - | - | - | - | - | - | - | - | no | - | - | no |
| A/Omsk/120/2018 | C1 | 30.01.2018 | complete | N | T | T | K | G | K | T | F | L | R | D | I | N | T | N | I | T | S | Q | R | S | E | T | R | no | - | yes | no |
| A/Kamchatka/1/2018 | C1 | 26.02.2018 | complete | N | T | T | K | G | S | T | F | L | R | D | I | N | T | S | I | T | S | Q | R | S | E | T | R | no | - | - | no |
| A/Krasnoyarsk/27/2018 | C1 | 24.03.2018 | complete | N | T | T | K | G | S | T | F | L | R | D | I | N | T | S | I | T | S | Q | R | S | E | T | R | no | - | - | no |
| A/Irkutsk/1965/2018 | C1 | 30.03.2018 | complete | N | T | T | K | G | S | T | F | L | R | D | I | N | T | S | I | T | S | Q | R | S | E | T | R | no | - | yes | no |
| A/Rostov-on-Don/1838/2018 | Original* | 25.05.2018 | partial | N | T | K | T | K | R | T | F | L | - | - | - | N | T | S | - | - | N | R | R | S | E | T | R | no | yes | yes | - |
| A/Rostov-on-Don/1839/2018 | Original* | 25.05.2018 | partial | N | T | K | T | K | R | T | F | L | R | D | I | N | T | S | - | T | N | R | R | S | E | T | R | no | yes | - | - |
