# Supplementary material for: Severe cases of seasonal influenza in Russia in 2017-2018
Source: PLoS One. 2019 Jul 29;14(7):e0220401. doi: 10.1371/journal.pone.0220401 (PMC6663013; doi:10.1371/journal.pone.0220401)
Supplement: S4 Table — (DOC) [file pone.0220401.s008.doc]

**S4 Table. Description of amino acid substitutions detected in A(H3N2) viruses.**

H3 numbering is used. Analysis was done using FluSurver ([http://flusurver.bii.a-star.edu.sg](http://flusurver.bii.a-star.edu.sg/)).

| **Gen** | **Mutation** | **Description** |
| --- | --- | --- |
| HA | N122D | Antigenic drift, removes a potential N-glycosylation site. |
| HA | T128A | Removes a potential N-glycosylation site. |
| HA | T131K | Antigenic drift. |
| HA | T135K | Removes a potential N-glycosylation site. |
| HA | T135N | Creates a potential N-glycosylation site. |
| HA | R142G | Antigenic drift. |
| HA | S144K | Antigenic drift. |
| HA | K160T | Creates a potential N-glycosylation site, antigenic drift. |
| HA | F193S | Antigenic drift. |
| HA | P194L | Antibody recognition site. |
| NA | R150S | Antigenic drift. |
| NA | D221N | Antigenic drift. |
| NA | I222V | In combination with E119V, increases drug resistance to oseltamivir. In A(H1N1), associated with mild increase in drug resistance to oseltamivir (less than 10 times). |
| NA | S245N | Creates a potential N-glycosylation site. |
| NA | S247T | Deletion 245-248 was associated with medium drug resistance. |
| NA | N329S | Antigenic drift, removes a potential N-glycosylation site. |
| PB2 | V63I | Mutation in this position correlated with changes in virulence. |
| PB2 | I588T | Host specificity shift (statistical, I – human, A – avian). |
| PB1 | S375N | Mutation in this position correlated with changes in virulence. |
| PA | Q57R | Host specificity shift (statistical, Q – human, R – avian). |
| PA | R266G | Mutation in this position correlated with changes in virulence. |
| NS1 | S87P | Host specificity shift (statistical, S – human, P – avian). |
| NS1 | E221K | Mutation in this position correlated with changes in virulence. |
| NS1 | T225A | Mutation in this position correlated with changes in virulence. |
| NS1 | R227K | Mutation in this position correlated with changes in virulence. |
