# Supplementary material for: Severe cases of seasonal influenza in Russia in 2017-2018
Source: PLoS One. 2019 Jul 29;14(7):e0220401. doi: 10.1371/journal.pone.0220401 (PMC6663013; doi:10.1371/journal.pone.0220401)
Supplement: S5 Table — (DOC) [file pone.0220401.s009.doc]

**S5 Table. Type B influenza virus cases characteristics.** (-) in case characteristics data (pneumonia diagnosis, risk group, vaccination) indicates that data is not available. (*) indicates that MDCK isolate was not available.

| Virus | Passage history | Collection date | Genome sequence | Fatal case | Pneumonia diagnosis | Risk group (WHO) | Vaccination |
| --- | --- | --- | --- | --- | --- | --- | --- |
| **B/Yamagata-like** |  |  |  |  |  |  |  |
| B/Murmansk/312/2018 | Original* | 2.4.2108 | partial | yes | yes | - | yes |
| B/Maykop/9868/2018 | Original* | 18.4.2018 | partial | yes | - | - | no |
| B/Birobidzhan/497/2018 | C1 | 24.3.2018 | complete | yes | yes | yes | - |
| B/Yekaterinburg/423/2018 | C1 | 13.04.2018 | complete | yes | - | yes | - |
| B/Ryazan/2/2018 | C1 | 3.4.2018 | complete | yes | yes | - | - |
| B/Chita/808/2018 | Original* | 19.2.2018 | partial | yes | - | yes | yes |
| B/Chita/807/2018 | Original* | 20.2.2018 | partial | yes | yes | yes | no |
| B/Nizhny Novgorod/4261/2018 | Original* | 11.4.2018 | partial | yes | - | yes | no |
| B/Dagestan/417/2018 | C1 | 5.4.2018 | complete | no | yes | yes | no |
| B/Tyva/4/2018 | C1 | 2.2.2018 | complete | no | - | yes | yes |
| B/Birobidzhan/479/2018 | Original* | 24.3.2018 | partial | no | - | - | yes |
| B/Birobidzhan/450/2018 | Original* | 22.3.2018 | complete | no | - | - | yes |
| B/Saint-Petersburg/3002/2017 | Original* | 6.12.2017 | complete | no | - | - | no |
| B/Mari El/1/2018 | Original* | 9.1.2018 | complete | no | - | - | no |
| B/Irkutsk/945/2017 | Original* | 30.10.2017 | partial | no | - | yes | - |
| B/Cherkessk/213/2018 | Original* | 24.4.2018 | partial | no | yes | yes | no |
| B/Cherkessk/211/2018 | Original* | 23.4.2018 | partial | no | yes | - | no |
| B/Primorie/28/2018 | C1 | 4.1.2018 | complete | no | - | yes | no |
| **В/Victoria-like** |  |  |  |  |  |  |  |
| B/Kaliningrad/310/2018 | C1 | 25.1.2018 | complete | no | - | yes | - |
| B/Kaliningrad/313/2018 | C1 | 25.1.2018 | partial | no | yes | - | - |
