# Supplementary material for: Correction: Severe cases of seasonal influenza in Russia in 2017-2018
Source: PLoS One. 2019 Dec 27;14(12):e0227382. doi: 10.1371/journal.pone.0227382 (PMC6934315; doi:10.1371/journal.pone.0227382)
Supplement: S6 Table — (DOCX) [file pone.0227382.s001.docx]

**S6 Table. Case characteristics and properties of influenza viruses isolated and characterized in the 2017-2018 epidemic season.** The table includes data on 87 isolated influenza viruses (42 of which were sequenced) and 33 influenza viruses (sequenced directly from clinical material).

| **#** | **Region** | **Samples**  **(viral isolates**  **##1-87;**  **original material ##88-120)** | | **Sex** | **Age** | **Vaccina**  **tion (date of vaccination if available)** | **Beginning of the disease** | | **Pre-period risk factors** | **Type/subtype** | **Virus (name is given for sequenced viruses, empty spaces indicate that sequencing was not done)** | **Oseltamivir**  **IC50, нМ** | **Zanamivir**  **IC50, нМ** |
| --- | --- | --- | --- | --- | --- | --- | --- | --- | --- | --- | --- | --- | --- |
| **1** | **Samara** | autopsy | | m | 15 | no data | no data | | no data | A(H1N1)pdm09 | A/Samara/117868/  2018 | **Resistant** **97.60** | 0.53 |
| **2** | **Astrakhan** | autopsy | | f | 30 | no | 23.02.18 | | no data | A(H1N1)pdm09 | A/Astrakhan/4/2018 | 0.27 | 0.47 |
| **3** | **Kemerovo** | autopsy and  nasopharyngeal swabs | | m | 65 | no | 28.02.2018 | | Pyelonephritis | A(H1N1)pdm09 | A/Kemerovo/4/2018 | 0.25 | 0.48 |
| **4** | **Kaliningrad** | autopsy and  nasopharyngeal swabs | | f | 35 | no data | 22.02.2018 | | no data | A(H1N1)pdm09 | A/Kaliningrad/3660/2018 | 0.29 | 0.35 |
| **5** | **Ulyanovsk** | autopsy | | m | 50 | no | 07.03.2018 | | Arterial hypertension | A(H1N1)pdm09 | A/Ulyanovsk/205/2018 | 0.19 | 0.63 |
| **6** | **Irkutsk** | autopsy | | m | 63 | yes  28.09.17 | 21.03.2018 | | Multiple sclerosis | A(H1N1)pdm09 | A/Irkutsk/1727/2018 | 0.25 | 0.41 |
| **7** | **Irkutsk** | autopsy | | m | 32 | no | 16.03.2018 | | no data | A(H1N1)pdm09 | A/Irkutsk/1728/2018 | 0.38 | 0.65 |
| **8** | **Birobidgan** | autopsy | | f | 70 | no data | 19.03.2018 | | no data | B/Yamagata | B/Birobidzhan/497/2018 | 4.94 | 4.57 |
| **9** | **Kaliningrad** | autopsy | | f | 58 | no data | 12.03.18 | | no data | A(H1N1)pdm09 | A/Kaliningrad/3860/2018 | 0.22 | 0.38 |
| **10** | **Kaliningrad** | nasopharyngeal swabs | | m | 35 | no data | 12.03.18 | | no data | A(H1N1)pdm09 | A/Kaliningrad/1146/2018 | 0.33 | 0.33 |
| **11** | **Ryazan** | autopsy and  nasopharyngeal swabs | | m | 63 | no data | 31.03.2018 | | no data | B/Yamagata | B/Ryazan/2/2018 | 5.21 | 4.15 |
| **12** | **Tomsk** | autopsy | | m | 28 | no data | 30.03.18 | | no data | A(H1N1)pdm09 | A/Tomsk/6301/2018 | 0.17 | 0.52 |
| **13** | **Yaroslavl** | autopsy | | m | no data | no data | no data | | no data | A(H1N1)pdm09 | A/Yaroslavl/8/2018 | 0.25 | 0.33 |
| **14** | **Omsk** | autopsy | | m | 52 | no | 03.04.2018 | | Polymyositis, chronic opisthorchiasis, chronic bronchitis | A(H3N2) | A/Omsk/680/2018 | 0.07 | 0.32 |
| **15** | **Sverdlovsk** | autopsy | | f | 2 | no data | no data | | no data | B/Yamagata | B/Yekaterinburg/423/2018 | 4.28 | 1.98 |
| **16** | **Krasnoyarsk** | autopsy | | f | 41 | no | no data | | no data | A(H1N1)pdm09 | A/Krasnoyarsk/8/2018 | 0.24 | 0.49 |
| **17** | **Vladimir** | autopsy | | m | 45 | no | 29.03.2018 | | Encephalopathy, Fatty degeneration of the liver | A(H1N1)pdm09 | A/Vladimir/263/2018 | 0.18 | 0.42 |
| **18** | **Vladimir** | autopsy | | m | 58 | no | 07.04.2018 | | COPD | A(H1N1)pdm09 | A/Vladimir/314/2018 | 0.19 | 0.43 |
| **19** | **Birobidgan** | autopsy | | m | 1 | no data | 07.05.18 | | no data | A(H1N1)pdm09 | A/Birobidzhan/1219/2018 | 0.18 | 0.36 |
| **20** | **Sverdlovsk** | autopsy | | m | 8 | no data | no data | | no data | A(H1N1)pdm09 | A/Yekaterinburg/472/2018 | 0.91 | 2.71 |
| **21** | **Krasnoyarsk** | autopsy | | m | 53 | no | no data | | no data | A/H1N1pdm09 |  | 0.26 | 0.52 |
| **22** | **Krasnoyarsk** | nasopharyngeal swabs | m | | 7 | no data | 23.08.2017 | no data | | A(H3N2) |  | 0.16 | 0.63 |
| **23** | **Rostov-on-Don** | nasopharyngeal swabs | f | | 30 | no data | 17.09.2017 | Pregnancy | | A(H1N1)pdm09 | A/Rostov-on-Don/3171/2017 | 0.26 | 0.49 |
| **24** | **Rostov-on-Don** | nasopharyngeal swabs | m | | 5 | no data | 20.09.2017 | no data | | A(H1N1)pdm09 | A/Rostov-on-Don/3196/2017 | 0.27 | 0.52 |
| **25** | **Kursk** | nasopharyngeal swabs | m | | 13 | no | 19.11.2017 | no data | | A(H3N2) | A/Kursk/1V/2017 | 0.15 | 0.58 |
| **26** | **Krasnodar** | nasopharyngeal swabs | m | | 18 | no | 13.12.2017 | no data | | A(H1N1)pdm09 | A/Krasnodar/421/2017 | 0.24 | 0.47 |
| **27** | **Novosibirsk** | nasopharyngeal swabs | f | | 23 | no data | 24.12.2017 | Pregnancy | | A(H3N2) |  | 0.14 | 0.53 |
| **28** | **Primorsky kray** | nasopharyngeal swabs | no data | | 1.1 | no | 07.01.2018 | Chronical bronchitis | | B/Yamagata |  | 6.145725 | 2.951957 |
| **29** | **Primorsky kray** | nasopharyngeal swabs | m | | 5 | no | 03.01.2018 | Chronical bronchitis | | B/Yamagata | B/Primorie/28/2018 | 6.808399 | 2.552586 |
| **30** | **Primorsky kray** | nasopharyngeal swabs | m | | 1.2 | no | 04.01.2018 | no data | | B/Yamagata |  | 6.599545 | 2.753698 |
| **31** | **Primorsky kray** | nasopharyngeal swabs | m | | 3 | no | 09.01.2018 | no data | | A(H1N1)pdm09 |  | 0.354956 | 0.596501 |
| **32** | **Novosibirsk** | nasopharyngeal swabs | f | | 2 | no data | 02.01.2018 | no data | | A(H3N2) |  | 0.11 | 0.53 |
| **33** | **Vladimir** | nasopharyngeal swabs | m | | 1.5 | no | 14.01.2018 | no data | | A(H1N1)pdm09 |  | 0.34 | 0.62 |
| **34** | **Primorsky kray** | nasopharyngeal swabs | m | | 63 | no data | no data | no data | | A(H1N1)pdm09 |  | 0.35504 | 0.526914 |
| **35** | **Ryazan** | nasopharyngeal swabs | f | | 82 | no | 19.01.2018 | Coronary heart disease | | B/Yamagata |  | 9.416577 | 3.268717 |
| **36** | **Irkutsk** | nasopharyngeal swabs | f | | 25 | no data | no data | Pregnancy | | B/Yamagata |  | 8.820376 | 3.694885 |
| **37** | **Samara** | nasopharyngeal swabs | m | | 26 | no data | no data | no data | | A(H1N1)pdm09 |  | 0.32 | 0.66 |
| **38** | **Tyva** | nasopharyngeal swabs | f | | 79 | yes | no data | no data | | B/Yamagata | B/Tyva/4/2018 | 6.36 | 2.51 |
| **39** | **Tyva** | nasopharyngeal swabs | m | | 15 | no | no data | no data | | B/Yamagata |  | 6.70 | 2.17 |
| **40** | **Tyumen** | nasopharyngeal swabs | f | | 7 | yes  03.10.17 | 15.01.2018 | no data | | A(H1N1)pdm09 | A/Tyumen/1/2018 | 0.19 | 0.32 |
| **41** | **Kaliningrad** | nasopharyngeal swabs | m | | 5 | no data | 24.01.2018 | no data | | B/Victoria | B/Kaliningrad/310/2018 | 4.18 | 4.77 |
| **42** | **Kaliningrad** | nasopharyngeal swabs | no data | | 37 | no data | 23.01.2018 | no data | | B/Victoria | B/Kaliningrad/313/2018 | 4.54 | 4.74 |
| **43** | **Kaliningrad** | nasopharyngeal swabs | no data | | 6 | no data | 20.01.2018 | no data | | A(H1N1)pdm09 |  | 0.26 | 0.55 |
| **44** | **Omsk** | nasopharyngeal swabs | f | | 66 | no | 29.01.2018 | no data | | A(H3N2) | A/Omsk/120/2018 | 0.08 | 0.31 |
| **45** | **Tomsk** | nasopharyngeal swabs | m | | 19 | no data | no data | no data | | B/Yamagata |  | 6.90 | 2.72 |
| **46** | **Khabarovky kray** | nasopharyngeal swabs | no data | | 11 | no data | 22.01.2018 | no data | | B/Yamagata |  | 6.12 | 2.35 |
| **47** | **Khabarovky kray** | nasopharyngeal swabs | no data | | 65 | no data | 19.01.2018 | no data | | B/Yamagata |  | 6.91 | 2.13 |
| **48** | **Belgorod** | nasopharyngeal swabs | f | | 56 | no data | 31.01.2018 | no data | | B/Yamagata |  | 8.70 | 3.25 |
| **49** | **Belgorod** | nasopharyngeal swabs | f | | 11 | no data | 05.02.2018 | no data | | B/Yamagata |  | 5.99 | 2.74 |
| **50** | **Kemerovo** | nasopharyngeal swabs | m | | 1 | no | 24.01.2018 | no data | | A(H1N1)pdm09 |  | 0.18 | 0.37 |
| **51** | **Kemerovo** | nasopharyngeal swabs | m | | 2 | no | 31.01.2018 | no data | | A(H1N1)pdm09 |  | 0.24 | 0.39 |
| **52** | **Kemerovo** | nasopharyngeal swabs | f | | 2 | no | 26.01.2018 | no data | | A(H1N1)pdm09 |  | 0.19 | 0.35 |
| **53** | **Kemerovo** | nasopharyngeal swabs | m | | 1 | no | 04.02.2018 | no data | | A(H1N1)pdm09 |  | 0.13 | 0.32 |
| **54** | **Kemerovo** | nasopharyngeal swabs | m | | 4 | no | 02.02.2018 | no data | | A(H1N1)pdm09 |  | 0.19 | 0.36 |
| **55** | **Komi** | nasopharyngeal swabs | f | | 26 | no | 06.02.2018 | Pregnancy | | A(H1N1)pdm09 |  | 0.34 | 0.65 |
| **56** | **S.-Petersburg** | nasopharyngeal swabs | m | | 14 | yes | 09.01.2018 | no data | | A(H3N2) | A/Saint Petersburg/646/2018 | 0.14 | 0.49 |
| **57** | **Vladikavkaz** | nasopharyngeal swabs | m | | 7 | no data | 04.02.2018 | no data | | A(H1N1)pdm09 |  | 0.36 | 0.57 |
| **58** | **Altay** | nasopharyngeal swabs | m | | 62 | no data | no data | no data | | B/Yamagata |  | 7.72 | 2.37 |
| **59** | **Altay** | nasopharyngeal swabs | f | | 19 | no data | no data | no data | | A(H1N1)pdm09 |  | 0.33 | 0.65 |
| **60** | **Adygeya** | nasopharyngeal swabs | m | | 3 | yes  26.10.17 | no data | no data | | A(H1N1)pdm09 | A/Mykop/2353/2018 | 0.15 | 0.32 |
| **61** | **Khanty-Mansiysk** | nasopharyngeal swabs | m | | 60 | no | 17.01.18 | COPD, Coronary heart disease | | B/Yamagata |  | 10.00 | 2.76 |
| **62** | **Kamchatka** | nasopharyngeal swabs | no data | | 29 | no | 23.02.2018 | no data | | A(H3N2) | A/Kamchatka/1/2018 | 0.17 | 0.64 |
| **63** | **Kamchatka** | nasopharyngeal swabs | f | | 63 | no | 22.02.2018 | no data | | A(H1N1)pdm09 |  | 0.27 | 0.50 |
| **64** | **Kamchatka** | nasopharyngeal swabs | f | | 52 | no | 24.02.2018 | no data | | B/Yamagata |  | 5.68 | 2.55 |
| **65** | **Kamchatka** | nasopharyngeal swabs | f | | 72 | no | 23.02.2018 | no data | | B/Yamagata |  | 6.85 | 1.71 |
| **66** | **Astrakhan** | nasopharyngeal swabs | f | | 22 | no data | 26.01.2018 | Pregnancy | | A(H1N1)pdm09 |  | 0.29 | 0.53 |
| **67** | **Astrakhan** | nasopharyngeal swabs | f | | 33 | no data | 11.02.2018 | Pregnancy | | A(H1N1)pdm09 |  | 0.28 | 0.50 |
| **68** | **Astrakhan** | nasopharyngeal swabs | f | | 19 | yes  27.10.17 | 18.02.2018 | Pregnancy | | A(H1N1)pdm09 | A/Astrakhan/9/2018 | 0.26 | 0.60 |
| **69** | **Khakasiya** | nasopharyngeal swabs | f | | 41 | yes  12.09.17 | no data | no data | | A(H3N2) | A/Abakan/249/2018 | 0.05 | 0.30 |
| **70** | **Krasnoyarsky kray** | nasopharyngeal swabs | f | | 30 | no | 15.03.2018 | no data | | A(H1N1)pdm09 |  | 0.30 | 0.57 |
| **71** | **Irkutsk** | nasopharyngeal swabs | f | | 31 | no | 10.03.2018 | Pregnancy | | B/Yamagata |  | 4.13 | 4.23 |
| **72** | **Irkutsk** | nasopharyngeal swabs | m | | 2 | no | 17.03.2018 | no data | | A(H1N1)pdm09 | A/Irkutsk/1615/2018 | 0.40 | 0.38 |
| **73** | **Irkutsk** | nasopharyngeal swabs | f | | 17 | no | 15.03.2018 | Pregnancy  HIV-I | | B/Yamagata |  | 14.38 | 2.58 |
| **74** | **Kaliningrad** | nasopharyngeal swabs | no data | | 16 | yes  17.10.17 | 12.03.2018 | no data | | A(H3N2) | A/Kaliningrad/1053/2018 | 0.06 | 0.30 |
| **75** | **Kurgan** | nasopharyngeal swabs | m | | 7 | no data | no data | no data | | B/Yamagata |  | 6.06 | 1.53 |
| **76** | **Kurgan** | nasopharyngeal swabs | f | | 6 | no data | no data | no data | | A(H1N1)pdm09 |  | 0.25 | 0.41 |
| **77** | **Dagestan** | nasopharyngeal swabs | f | | 73 | no | 02.04.2018 | no data | | B/Yamagata | B/Dagestan/417/2018 | 6.63 | 1.63 |
| **78** | **Dagestan** | nasopharyngeal swabs | f | | 19 | no | 02.04.2018 | Pregnancy | | B/Yamagata |  | 6.25 | 1.73 |
| **79** | **Dagestan** | nasopharyngeal swabs | m | | 7 | no | 06.04.2018 | no data | | A(H1N1)pdm09 |  | 0.21 | 0.41 |
| **80** | **Irkutsk** | nasopharyngeal swabs | f | | 28 | no | 29.03.2018 | Pregnancy | | A(H3N2) | A/Irkutsk/1965/2018 | 0.07 | 0.44 |
| **81** | **Irkutsk** | nasopharyngeal swabs | f | | 29 | no | no data | Pregnancy | | A(H1N1)pdm09 |  | 0.22 | 0.45 |
| **82** | **Krasnoyarsky kray** | nasopharyngeal swabs | f | | 31 | no | 23.03.2018 | Pregnancy | | A(H3N2) | A/Krasnoyarsk/27/2018 | 0.10 | 0.40 |
| **83** | **Dagestan** | nasopharyngeal swabs | f | | 32 | no | 16.04.2018 | no data | | A(H1N1)pdm09 | A/Dagestan/602/2018 | 0.21 | 0.42 |
| **84** | **Dagestan** | nasopharyngeal swabs | f | | 28 | no | 09.04.2018 | Pregnancy | | A(H1N1)pdm09 |  | 0.20 | 0.43 |
| **85** | **Krasnoyarsky kray** | nasopharyngeal swabs | f | | 20 | no data | 29.03.2018 | Pregnancy | | B/Yamagata |  | 5.78 | 2.24 |
| **86** | **Krasnoyarsky kray** | nasopharyngeal swabs | f | | 29 | no | 26.04.2018 | Pregnancy | | A(H3N2) |  | 0.07 | 0.30 |
| **87** | **Krasnoyarsky kray** | nasopharyngeal swabs | m | | 14 | no | 29.04.2018 | no data | | A(H1N1)pdm09 |  | 0.28 | 0.42 |
| **88** | Irkutsk | Original  autopsy | m | | 37 | no | 17.03.2018 | Obesity | | A(H1N1)pdm09 | A/Irkutsk/1648/2018 | | |
| **89** | Irkutsk | Original  autopsy | f | | 41 | no | 12.03.2018 | no data | | A(H1N1)pdm09 | A/Irkutsk/1649/2018 | | |
| **90** | Krasnoyarsky kray | Original  autopsy | m | | 53 | no | no data | no data | | A(H1N1)pdm09 | A/Krasnoyarsk/54/2018 | | |
| **91** | Zabaykalsky kray | Original  autopsy | m | | 63 | no | 26.01.2018 | COPD | | A(H3N2) | A/Chita/673/2018 | | |
| **92** | Zabaykalsky kray | Original  autopsy | m | | 10 | yes  11.09.2017 | 16.02.2018 | Congenital Heart Anomaly | | B/Yamagata | B/Chita/808/2018 | | |
| **93** | Zabaykalsky kray | Original  autopsy | m | | 82 | no | 10.02.2018 | COPD | | B/Yamagata | B/Chita/807/2018 | | |
| **94** | Murmansk | Original  autopsy | f | | 15 | 02.10.17 yes | 26.03.2018 | no data | | B/Yamagata | B/Murmansk/312/2018 | | |
| **95** | Nizhny Novgorod | Original  autopsy | m | | 3 | no | 03.04.2018 | no data | | B/Yamagata | B/Nizhny Novgorod/4261/2018 | | |
| **96** | Adygeya | Original  autopsy | f | | 64 | no | no data | no data | | B/Yamagata | В/Maykop/9868/2018 | | |
| **97** | Irkutsk | Original nasopharyngeal swab | f | | 3 | no data | 11.12.2017 | no data | | А(H1N1)pdm09 | A/Irkutsk/42/2017 | | |
| **98** | Samara | Original nasopharyngeal swab | m | | 64 | no data | 02.01.18 | no data | | A/H1N1swine | A/Samara/823/2018 | | |
| **99** | Krasnoyarsky kray | Original nasopharyngeal swab | m | | 7 | no data | 24.08.2017 | no data | | A(H3N2) | A/Krasnoyarsk/16266/2017 | | |
| **100** | Saint-Petersburg | Original nasopharyngeal swab | m | | 25 | no | 21.09.2017 | no data | | A(H3N2) | A/Saint-Petersburg/2227/201 | | |
| **101** | Nizhny Novgorod | nasopharyngeal swabs | m | | 52 | no | 21.09.2017 | no data | | A(H3N2) | A/Nizhny Novgorod/8320/2017 | | |
| **102** | Sakha | Original nasopharyngeal swab | m | | 90 | yes 06.10.17 | 23.10.17 | no data | | A(H3N2) | A/Sakha/1566/2017 | | |
| **103** | Sakha | Original nasopharyngeal swab | f | | 79 | no | 23.10.17 | no data | | A(H3N2) | A/Sakha/1567/2017 | | |
| **104** | Moscow | Original nasopharyngeal swab | m | | 59 | no | 25.10.2017 | no data | | A(H3N2) | A/Moscow/1/2017 | | |
| **105** | Novy Urengoy | Original nasopharyngeal swab | f | | 31 | no | 31.10.2017 | no data | | A(H3N2) | A/Novy Urengoy/2118/2017 | | |
| **106** | Irkutsk | Original nasopharyngeal swab | f | | 2 | no data | 28.10.2017 | no data | | A(H3N2) | A/Irkutsk/1016/2017 | | |
| **107** | Irkutsk | Original nasopharyngeal swab | f | | 4 | no data | 28.10.2017 | no data | | A(H3N2) | A/Irkutsk/1017/2017 | | |
| **108** | Kamchatka | Original nasopharyngeal swab | m | | 5 | no | 08.12.2017 | no data | | A(H3N2) | A/Kamchatka/414/2017 | | |
| **109** | Novosibirsk | Original nasopharyngeal swab | f | | 23 | no | 24.12.2017 | Pregnancy | | A(H3N2) | A/Novosibirsk/265/2017 | | |
| **110** | Samara | Original nasopharyngeal swab | f | | 21 | no data | 08.01.2-18 | no data | | A(H3N2) | A/Samara/829/2018 | | |
| **111** | Khabarovskiy kray | Original nasopharyngeal swab | no data | | 6 | no | 10.01.2018 | no data | | A(H3N2) | A/Khabarovsk/31/2018 | | |
| **112** | Rostov-on-Don | Original nasopharyngeal swab | m | | 4 | no data | 25.05.2018 | no data | | A(H3N2) | A/Rostov-on-Don/1838/2018 | | |
| **113** | Rostov-on-Don | Original nasopharyngeal swab | m | | 10 | no data | 25.05.2018 | no data | | A(H3N2) | A/Rostov-on-Don/1839/2018 | | |
| **114** | Irkutsk | Original nasopharyngeal swab | f | | 1 | no data | 30.10.2017 | no data | | B/Yamagata | B/Irkutsk/945/2017 | | |
| **115** | Saint-Petersburg | Original nasopharyngeal swab | m | | 26 | no | 06.12.2017 | no data | | B/Yamagata | B/Saint-Petersburg/3002/2017 | | |
| **116** | Mari El | Original nasopharyngeal swab | f | | 36 | no | 09.01.2018 | no data | | B/Yamagata | B/Mari El/1/2018 | | |
| **117** | Birobidzhan | Original nasopharyngeal swab | no data | | 45 | yes | 22.03.2018 | no data | | B/Yamagata | B/Birobidzhan/450/2018 | | |
| **118** | Birobidzhan | Original nasopharyngeal swab | no data | | 30 | yes | 24.03.2018 | no data | | B/Yamagata | B/Birobidzhan/479/2018 | | |
| **119** | Cherkessk | Original nasopharyngeal swab | m | | 63 | no | 23.04.2018 | no data | | B/Yamagata | B/Cherkessk/211/2018 | | |
| **120** | Cherkessk | Original nasopharyngeal swab | m | | 86 | no | 24.04.2018 | no data | | B/Yamagata | B/Cherkessk/213/2018 | | |
